# Results of a cluster randomised controlled trial to reduce risky use of alcohol, alcohol-related HIV risks and improve help-seeking behaviour among safety and security employees in the Western Cape, South Africa

**DOI:** 10.1186/s13011-015-0014-5

**Published:** 2015-05-08

**Authors:** Nadine Harker Burnhams, Leslie London, Ria Laubscher, Elmarie Nel, Charles Parry

**Affiliations:** Alcohol, Tobacco and Other Drug Research Unit, South African Medical Research Council, P.O. Box 19070, Cape Town, 7505 South Africa; School of Public Health and Family Medicine, Falmouth Building, Faculty of Health Sciences University of Cape Town, Cape Town, South Africa; Biostatistics Unit, South African Medical Research Council, P.O. Box 19070, Cape Town, 7505 South Africa; Department of Psychiatry, Stellenbosch University, P.O. Box 19063, Cape Town, Tygerberg South

**Keywords:** Alcohol, Employees, Prevention, Alcohol-related HIV risks, Evidence-based, Team awareness

## Abstract

**Objective:**

To test the effectiveness of a programme aimed at reducing the risky use of alcohol and alcohol-related HIV risk and increase help-seeking behaviour among a sample of municipal employees in the Western Cape Province, South Africa.

**Methods:**

A clustered randomised controlled trial was conducted in 2011–2012 among 325 employees. The eight hour intervention, Team Awareness (TA), addressing behavioural risk among employees was administered to 168 employees in the intervention arm and the 157 employees in the control arm who received a one-hour wellness talk.

**Results:**

The results show that TA had the greatest impact on risky drinking practices and hangover effects. There was a significant group × time interaction (F (1, 117) = 25.16, p < 0.0001) with participants in the intervention condition reducing number of days on which they engaged in binge drinking. There was also a significant time effect with participants in the intervention condition reducing the likelihood of going to work with a hangover (F (1,117) = 4.10, p = 0.045). No reduction in HIV-related risk behaviours were found.

**Conclusions:**

This intervention study was able to demonstrate a modest but significant reduction in risky drinking practices and hangover effects. This provides encouraging evidence for the effectiveness of interventions that address risky use of alcohol among employed persons, further providing a launch pad for strengthening and replicating future RCT studies on workplace prevention, especially in developing country settings.

**Clinical Trial Registration Number:**

Pan-African Control Trial Registry (201301000458308).

## Introduction

The risky use of alcohol by employed persons has generated considerable global discussion [1] since it holds negative implications for the health of employees and ultimately impacts on productivity through poor work performance [2], high absenteeism rates, presenteeism, accidents and injuries [3-5]. Alcohol specifically has been identified as a leading risk factor for death and disability globally. The 2010 analysis of 67 risk factors and risk factor clusters for death and disability reported in the special issue of the Lancet [6] found that alcohol was the third leading risk factor for death and disability accounting for 5.5% of disability adjusted life years (DALYs) lost globally and the highest risk factor in sub-Saharan Africa. If left untreated, harmful alcohol use can exact substantial costs from employees and employers since hazardous use is a major risk factor for injury and communicable and non-communicable diseases [7,8]. Although the proportion of the population consuming alcohol in South Africa is low as compared to other developing countries, the proportion of the population who consume alcohol riskily, defined in this study as drinking 5 or more drinks for males and 3 or more drinks for women per day, is high and estimated to be 9.2% [9,10].

In South African workplaces problematic alcohol use has been found to be more prevalent among employees than problematic drug use [11]. For instance, in a survey of police officers in the Limpopo province, 55% of officers admitted to binge drinking [12]. Similarly, findings from a random sample of 325 employees employed within a municipality in the Western Cape Province found that more than half of the participants who reported consuming alcohol engaged in binge drinking (defined as days in past 30 days an employee had more than five drinks at one sitting) (76.1%) [13], a figure far higher than equivalent provincial estimates of past month binge drinking (defined as drinking five or more alcohol drinks on the same occasion on at least 1 day in the past 30 days or 7 days) of 16.3% in the South African National HIV, Behaviour and Health Survey [9].

Compounding high levels of risky alcohol use in South Africa is the problem of HIV/AIDS. A study of safety and security employees [13] found that alcohol users were significantly more likely to have one or more HIV risk exposures when compared to their non-using counterparts, thus increasing the risk for HIV infection [14,15]. Similar findings have been reported in a study of mine workers [16]. This is corroborated in a recent meta-analysis of 12 experimental studies which found that an increase in blood alcohol concentration of 0.1 mg/ml was associated with an increase of 2.9% in the likelihood of engaging in unprotected sex [17].

Over the last three decades, prevention efforts to reduce the adverse effects of risky alcohol use or drug use in workplace settings has become a priority for many organisations, government agencies and other constituencies [1,18-23]. Coupled to this has been the increased global focus on the importance of implementing and disseminating evidence-based interventions (EBIs) targeting alcohol or drug use [24,25]. A large body of literature supports the implementation of EBIs designed for use in the workplace since the workplace provides a) an environment for providing alcohol and drug prevention messages to working adults [19,23,26]; b) provides easier accessibility to risky users considering the length of time a worker spends at work [19,27]; and c) may also have trickle-down effects, suggesting that messages of prevention can be filtered through to the family of the employee [21]. While there are clear recommendations for the dissemination of prevention programmes in workplace settings, systematic reviews [19,28,29] of published literature on workplace alcohol or drug prevention programmes have highlighted the existence of few methodologically adequate studies of workplace interventions. According to these reviews, weaknesses largely relate to representativeness of samples, consent and participation rates, blinding, post-test time-frames, contamination and reliability, and validity of measures used. Despite these limitations the reviews unanimously conclude that brief interventions, interventions contained within health and life-style checks, psychosocial skills training and peer referral have the potential to be replicated and should be strengthened to produce beneficial results.

Unfortunately in South Africa, workplace alcohol and drug prevention initiatives have followed passive, unscientific practices that generally focus on imparting knowledge, but do not guarantee behaviour change [30,31]. In addition, such programmes tend not to focus on related risks such as HIV.

To address these gaps this study aimed to test the effectiveness of a programme aimed at reducing the risky use of alcohol (defined below) and alcohol-related HIV risk and increase help-seeking behaviour among a sample of employees. More specifically, the study hypothesised that participation in the intervention:

1. Would have reduced the number of days in the past 30 days the employee had ≥ 5 drinks on one occasion from Time 1 – Time 3.
2. Would be positively associated with a reduction in last 6 months going to work with a hangover from Time1 – Time 3.
3. Would be positively associated with a reduction in last 6 months call in sick episodes because of hangover from Time1 –Time 3.
4. Would have resulted in a reduction of problematic substance use as calculated by the CAGE scores from Time – Time 3.
5. Increased willingness to use EAP for a personal or work-related problem would have improved from Time1 – Time 2 and sustained at Time 3.
6. Would be associated with a positive change in team drinking climate from Time 1 – Time 2 – Time 3.
7. Increased group cohesion among employees from Time1 – Time 2 – Time 3.
8. Reduced employee exposure to multiple HIV risks from Time1 – Time 2 – Time 3.

## Methods

### Study design

A clustered randomised control trial was conducted in 2011–2012 to determine the impact of a behavioural prevention programme, Team Awareness (TA), for reducing risky drinking and associated HIV risks (primary outcomes) among employees, and enhancing aspects of the work group environment that support on-going prevention (secondary outcomes) at post-intervention (2 weeks) and three-month follow-up. A clustered design was opted for since TA sought to bring about changes to the workgroup.

### Study setting

The study was conducted within two safety and security divisions of a municipality in the Western Cape Province, South Africa. For the purposes of anonymity, the participating municipality, sector and divisions will not be named. One of the divisions generally responds to emergency situations and primarily protects society from all types of accidents and emergencies, whilst the second division works in partnership with communities to uphold law and order. The participating municipality was self-identified by a contact person of its Employee Assistance Programme (EAP), who acted as a broker, facilitating entry into the safety and security department and the two divisions.

### Description of study sample, selection and participants

At the time of this study the two divisions selected for participation in the study had a total of 1349 (of which 128 participated) and 615 (of which 197 participated) employees, respectively. All employees within the two divisions were eligible for participation in the study. Criteria for inclusion in the research study were: a) all employees participating in the study should be conversant in English, and b) should work within a team context. Intact workgroups (defined as a group of people who work together on a regular basis for a few months a year or longer) were randomly selected from each of these divisions to complete a pen and paper self-report questionnaire and to attend a programme intervention (TA). Intact workgroups were randomly assigned to either the intervention arm which consisted of the eight hour TA training session, or to the control arm which consisted of a one hour wellness session, using simple systematic randomisation. The randomisation processes for the two divisions were different due to the internal structure and setup of the divisions. More specifically, for the first division the randomisation process was layered. At the time of the study, there were 30 stations in the Cape Town Metropole. Individual stations consisted of at least three workgroups/platoons all working shifts. The first layer consisted of taking the population size of 30 stations and assigning a number 1–30 to each station. From this layer, 20 stations were randomly selected to be part of the research study. The second layer included randomising each of the 20 stations to a control (n = 10) or intervention condition (n = 10). Once completed, a third layer of randomisation took place; one of the three workgroups within each of the 20 randomly selected stations was selected to participate in the study.

The structure of the second division was different to that of the first. Although, they are also situated across the Cape Town Metropole the main offices are centralised to 4 areas of the metropole (North, South, East and West). Within each of the four Metropole main offices, are teams which service a certain geographical area. For instance, area South would be expected to service 2–4 geographical areas. Considering that one of the criteria for participation was being a member of a workgroup and that the sample size was set at 190 and 20 groups, all workers within these larger areas were eligible for participation and the areas were randomised into a control or intervention condition (North, South, East and West). The tossing of a coin was used to randomise the areas, and areas North and South became the control conditions and areas East and West became the intervention conditions.

The participating divisions were identified prior to the randomisation process. Participating divisions were asked to provide their shift rosters and work orders. Participating divisions and participants were not privy to the randomisation process, but were informed after the randomisation process on which groups would need to be away from work for possibly six hours (factoring in travelling time) and which workgroups would be away for one hour only (controls). Randomisation of clusters into a control or intervention arm was done by the principal investigator and a biostatistician.

Although the participants were not aware of whether they were assigned to an intervention or control condition, they were aware that they were assigned to either the one hour session or an eight hour (4 hour sessions at a time, over two weeks) session. The interventionists and project staff were not blinded. This was not possible as the interventionists worked mainly with those in the experimental arm of the study, and other fieldworkers were only involved with participants in the control arm. The interventionists and fieldworkers adhered strictly to study protocols and ethical principles, in respect of engaging with participants. The interventionists did not collect questionnaire data; this was the role of the fieldworkers.

To minimise possibilities for contamination, it was ensured that the TA and control interventions did not take place on the same day. Additionally the use of a clustered RCT in itself also assists in avoiding contamination [32] since groups rather than individuals are randomized to a programme [33]. Finally, the Cape Town Metropole is a large geographical area and the stations for both divisions are far apart, and stations rarely interact.

Responses were kept confidential and no individual data were given to management or the municipality. A total of 20 workgroups were selected for the intervention and 20 for the control arm of the study.

### Sample size

In the absence of prevalence data on the extent of alcohol use among safety and security employees, sample size calculation was based on the power as calculated for cluster randomised control trials. Calculations depend on two sample sizes (groups and individuals within groups), the intra-class correlation (ICC), and the effect size [34,35]. Based on an anticipated sample of 40 workgroups (with approximately 10 employees in each), of which, 20 were randomised to the intervention arm, and 20 to the control arm, with an estimated ICC of .03 (such that 3% of total variability in outcomes reflected workgroup differences), 190 employees were needed in each arm. With ICC = .03, the design yields an 80% power. Although there were no refusals to participate, due to circumstance beyond the control of the researchers, only 325 employees participated at baseline.

### Intervention design

**Figure 1:**
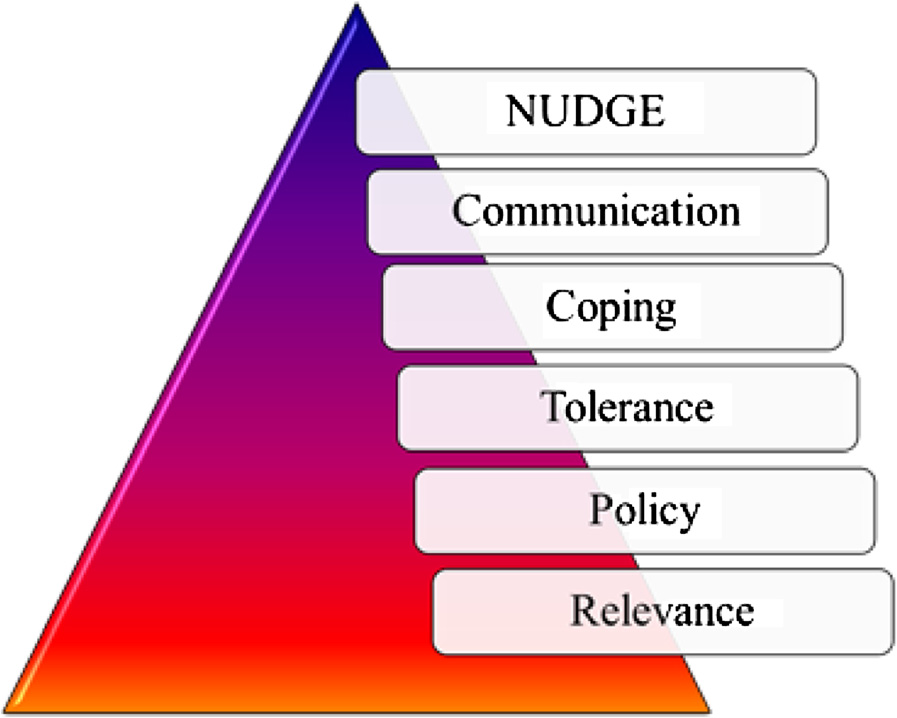
The six modules covered in the team awareness intervention.

The intervention was based on Team Awareness, an evidence-based workplace training programme developed by Texas Christian University (TCU), USA [36]. The programme addresses behavioural risks among employees, their co-workers and indirectly, their families. TA consists of six training modules (Figure 1) presented to employees over an eight-hour session. It aims to promote social interaction among work teams, promote a positive and healthy work and team environment, and facilitates the de-stigmatisation of help-seeking thus encouraging such proactive behaviours (see Figure 1 for a description of the 6 modules) [36,37]. TA has been scientifically evaluated in studies in the USA. The results from a study among municipal workers in the USA suggested that employees receiving the TA intervention significantly reduced problem drinking from 20% to 11% as compared to control subjects who showed no significant change at pre and post-test (13% respectively): F = 6.78, p = 0.01). Additionally, the study found significant reductions in working with a hangover or missing work because of a hangover from 16% to 6% as compared to control subjects who showed no change at pre and post-test (9% respectively) (F = 7.34, p = 0.007) [29]. The TA intervention was sourced via a systematic review and adapted for the local context, described extensively elsewhere [38]. TA sessions were conducted by locally recruited interventionists with appropriate higher education who underwent training by the TA programme developer over a period of a week with rigorous peer review. The intervention sessions were implemented over a period of 2 weeks at locations and work times convenient to the participating municipality. To monitor implementation fidelity, a system of completing facilitator session notes, debriefing sessions and spot visits were employed.

### Data collection process

Data were collected at baseline, post intervention (two weeks) and at three month follow-up, during work hours, using an 18 page self-completed structured Workplace Questionnaire (WQ). The interventions were conducted in English. Participation was voluntary and based on signed individual informed consent after randomisation. The study was approved by the Human Sciences Research Ethics Committee at the University of Cape Town.

### Measures

#### Alcohol consumption measures

Items examining alcohol consumption were drawn from instruments developed by the TCU Workplace Project [39], and from the South African Community Epidemiology Network on Drug Use (SACENDU) tool [40]. Questions elicited responses on past 30 day use of alcohol and; days in past 30 days a participant had five or more drinks at one sitting (“defined as binge drinking”). Participants were also asked whether in the last six months, they went to work with a hangover or called in sick because of a hangover. Likert scales coded 0 = ‘never’ to 4 = ‘daily or almost daily’ were used to reflect these drinking behaviours, The CAGE questionnaire, a self-report four-item test was used to screen for problematic drinking [41]. The cut-off score for CAGE is 2, which indicates symptoms of problematic drinking. For the purposes of this study, risky drinking was defined on the basis of binge drinking and/or a CAGE score of GE 2.

#### Alcohol-related HIV risk

Seven HIV risk questions were taken out of a 25 item questionnaire focusing on sexual thoughts, feelings and behaviours that patients recollected from the last time they were under the influence of a single psychoactive agent [42]. The seven questions selected, were the questions related to specifically the behaviour of employees when under the influence of a primary substance of abuse. Participants were asked to give a 0 = ‘No’ or 1 = ‘Yes’ response. Although the items in the questionnaire are of relevance and have good face validity, the psychometric properties of the instrument have not been formally established [42].

#### Workplace drinking climate

Scales used to measure Workplace drinking climate and group cohesion were developed by the Texas Christian University (TCU), Fort Worth, Texas. The TCU scales have all been previously validated in a study of municipal workers in the USA [43] but also within this sample by estimating Cronbach’s alpha statistics for each scale [13].

Drinking climate was assessed by the frequency of four co-worker behaviours. Responses ranged from 1 = ‘never’ to 5 = ‘almost always’. Reliability, as measured by Cronbach’s alpha was 0.74 [13].

#### Group cohesion

A 5-item measure of group cohesion was used. Employees rated each item along the same 5-point scale 1 = ‘strongly disagree’ to 5 = ‘strongly agree’. The average of the items was used as a composite measure of perceived group cohesiveness, which had good internal consistency as measured with Cronbach’s Alpha (α = 0.71). [13].

#### Employee Assistance Programme (EAP) utilisation

Thirteen items were used to assess knowledge on the existence of a workplace EAP service, willingness to access such a service as well as participant willingness to recommend the EAP to a co-worker. Responses to single items varied from ‘Yes-No’ answers to Likert scales [44]. The items do not form part of a scale.

### Statistical analysis

Data for the clustered randomised control trial was analysed using SAS/STAT software, Version 9.2. Data were analysed using a random effects model since data were hierarchical and clustered, and random effects models can be used to analyse data with clustered sources of variability. The SAS GLIMMIX (generalized linear mixed models) procedure was used since it fits statistical models to data with non-constant variability, and where responses are not normally distributed. It further addresses the hierarchical nature in different random statements. For analysis, entered into the model were group, time (refers to time1, time 2 and 3 month follow-up) and division. Considering that division was part of the sampling frame and emerged as a predictor for dropout, adjustment was taken care of in the model. A significant statistical group × time interaction on any of the variables measured, in the predicted direction, was indicative of the effect of the intervention over time. Gaussian, Binary and Poisson regressions were used depending on whether the outcome variable was continuous, categorical or a count variable.

Thus, analysis of the trial outcomes took into account stratification by division as two divisions participated in the study, repeated measures considering that participants were tested more than once (baseline, time 2 and 3-month follow-up), and clustering considering that actual intact workgroups were randomly assigned to an intervention or control arm. Effects of the intervention were estimated by comparing employees in the intervention and control arms, adjusting for clustering. Time was used as a discrete variable in the analysis, since time specific estimates were investigated, safeguarding against multiplicity of testing.

Prior to final analysis, participant attrition and the extent to which drop-out introduced bias, logistic regressions (univariate) on baseline variables to predict dropout was run, adjusted for clustering. A prediction model for completeness was developed using a stepwise logistic regression model. All baseline variables were entered into the model with a probability of 0.1 to stay in the model. Division and age emerged as predictors, although age was not a significant predictor. The law and order division were more likely to dropout when compared to emergency employees. This was accounted for in the final models, because division was one of the cluster variables. The predicted proportions from the stepwise model, division and age (although not statistically significant) were used to create a weight for completeness so that complete cases were weighted higher. For all significance testing, the F-statistic and p-values (<0.05 for statistical significance) are reported.

## Results

### Participation and comparability of samples

Of the 325 participants who participated in the study, 168 were randomly assigned to the intervention group, and 157 participants were in the control group. Table 1 provides a description of the baseline characteristics of employees within the control, and intervention conditions. Most participants (87%) were males. No statistically significant differences between the two groups were noted at baseline.

**Table 1:** **Baseline demographic and behaviour characteristics (N = 325)**

	***Intervention group*** ***(n = 168)***	***Control group*** ***(n = 157)***	***P-value***
**Women**	24	18	0.449
**Men**	144	138	
**Mean age**	41.78	36.48	0.376
***Standard deviation***	*21-57*	*21-60*	
**Education**			
Grade7-11	10	14	0.398
Grade 12	121	114	
Tertiary	33	24	
**Length of employment**			0.949
0-5 years	24	23	
5-10 years	79	72	
10-15 years	32	32	
More than 15 years	32	26	
**Marital status**			0.245
Single	33	39	
Married	118	96	
Divorced	16	16	
Widowed	0	2	
**Language**			0.521
Afrikaans	72	62	
English	46	52	
Xhosa	35	34	
Other	15	9	
**Past 30 day five or more drinks**	112	101	0.254
**CAGE scores**			0.304
0 (no risk)	69	70	
1	19	15	
2	12	9	
3	10	4	
4	3	4	
**Total**	113	102	
**Hangovers**	113	101	0.259
Never	81	66	
Less than monthly	20	20	
Monthly	8	10	
Weekly	4	4	
Daily or almost daily	0	1	

### Attrition

**Figure 2:**
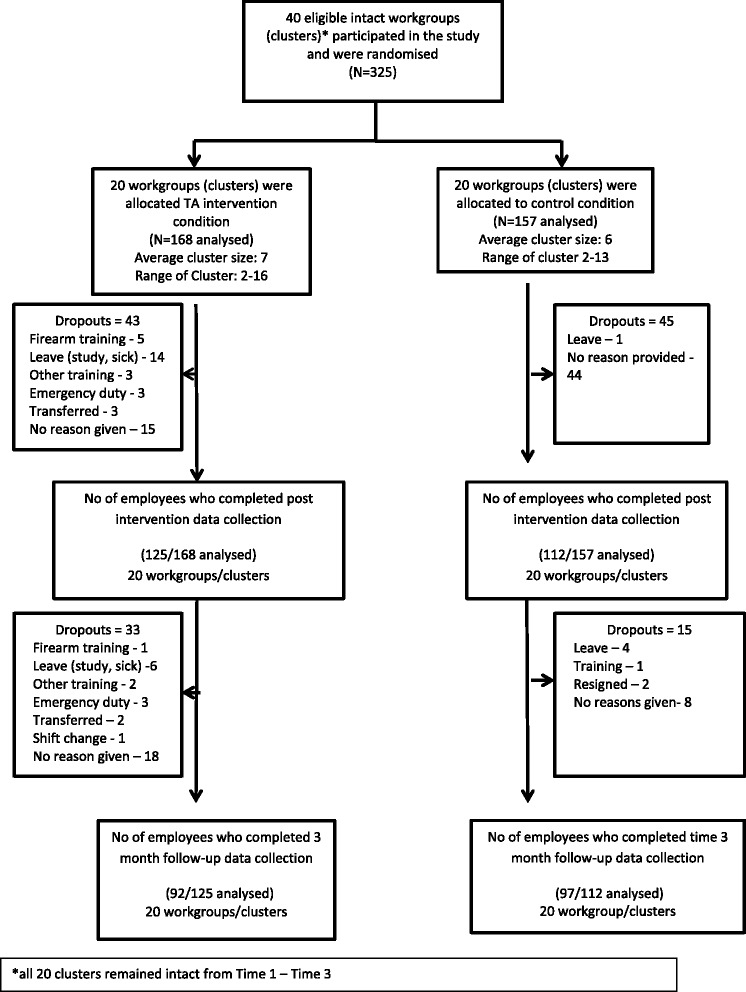
Flow diagram for cluster trial.

Two hundred and thirty seven of the 325 (73%) participants completed post-intervention and 189 of the 325 (58%) completed three-month follow-up assessment (see Figure 2). No intact clusters were lost in dropout. Only employees who completed post-intervention questionnaires were followed up at three months. There were no significant differences between those who completed the intervention and those lost through attrition (p > 0.05), except that division emerged as a significant predictor suggesting that participants in the law and order division were twice more likely to dropout when compared to their counterparts (OR = 2.73; p = < 0.001; 95% CI = 1.67 – 4.44).

### Days having 5 or more drinks at one sitting in the past 30 days

The results show that TA had the greatest impact on days having 5 or more drinks at one sitting in the past 30 days (Table 2). There was a group × time interaction *F* (1, 117) = 25.16, p < 0.0001, with participants in the intervention condition reducing the mean number of days having five or more drinks from 2.1 days to 1.4 days, in the predicted direction. Participants in the control arm increased on mean days having 5 or more drinks from 1.6 days at baseline to 2.1 days at 3 month follow-up.

**Table 2:** Risky alcohol use practices among municipal safety and security staff****

**Risky use of alcohol outcomes**	Group*	**Baseline**				**3-month follow-up**				**F-statistic**	**P-value**
		***N***	***Mean***	***SE***	***95% CI***	***N***	***Mean***	***SE***	***95% CI***		
*Binge drinking (Days having 5 or more drinks in past 30 days)*	I	136	0.070 (2.1 days)**	0.010	0.053-0.093	75	0.045 (1.4 days)**	0.007	0.033-0.062	**F(1,117) =25.16**	**P < 0.0001**
	C	121	0.052 (1.6 days)**	0.008	0.038-0.071	63	0.070 (2.1 days)**	0.012	0.050-0.097		
Going to work with a hangover***	I	136	0.233	0.041	0.161-0.324	75	0.129	0.041	0.067-0.234	F(1,117) =0.24	P = 0.626
	C	121	0.276	0.047	0.193-0.379	63	0.198	0.055	0.111-0.333		
Calling in sick as a result of a hangover***	I	136	0.095	0.029	0.051-0.169	75	0.046	0.023	0.017-0.121	F(1,117) =0.01	P = 0.905
	C	121	0.111	0.033	0.061-0.194	63	0.060	0.029	0.022-0.152		
*CAGE*	I	136	0.177	0.035	0.118-0257	75	0.099	0.035	0.048-0.194	(F1,117) =0.08	P = 0.773
	C	121	0.136	0.033	0.082-0.215	63	0.089	0.037	0.038-0.193		

*I = Intervention; C = Control.

**Corresponding reduction in days. Variable analysed as a count variable.

***Hangover variables showed no group × time effect but significant time effects (see Results section).

Bold interface signifies significant group × time effect.

****Of those who consume alcohol.

### Going to work with a hangover or calling in sick as a result of a hangover

There were no significant group × time interaction on the variable going to work with a hangover F (1,117) = 0.24, p = 0.626; or calling in sick with a hangover F (1, 117) = 0.01, p = 0.905 (Table 2). There was however a significant time effect with participants in both the intervention and control conditions reducing the likelihood of going to work with a hangover F (1,117) = 4.10, p = 0.045. In relation to calling in sick with a hangover, results suggest an effect of time, with borderline significance, such that participants in both the control and intervention condition reduced the likelihood of calling in sick because of a hangover F (1, 117) = 3.38, p = 0.068.

### CAGE scores

Although there was no significant group × time intervention effect F (1,117) =0.08, p = 0.773 (Table 2), the findings suggest a decrease in problematic alcohol abuse in the intervention group, in the expected direction. This effect was also observed in the control arm of the study.

### Workplace drinking climate

Although the results demonstrate an increase towards a workplace climate that is less favourable of drinking among those in the intervention arm in the right direction, the effect was not sustained at 3 month follow-up. Findings suggest no significant group × time effect F (2,413) = 2.15, p = 0.11) (Table 3).

**Table 3:** **Workplace drinking climate and group cohesion (baseline, post intervention and three-month follow-up)**

**Climate and cohesion**	Group*	**Baseline**				**Post intervention**				**3-month follow-up**				**F-statistic**	**P-value**
		**N**	**Mean**	**SE**	**CI**	**N**	**Mean**	**SE**	**CI**	**N**	**Mean**	**SE**	**CI**		
*Workplace drinking climate*	I	166	2.934	0.090	2.757-3.111	126	2.883	0.095	2.696-3.069	93	2.910	0.101	2.712-3.108	F (2,413) = 2.15	P = 0.11
	C	155	2.954	0.089	2.778-3.130	109	2.970	0.096	2.782-3.158	95	2.775	0.098	2.582-2.968		
*Group cohesion*	I	169	3.302	0.069	3.166-3.439	126	3.464	0.073	3.320-3.607	93	3.273	0.077	3.121-3.424	F (2,421) = 1.39	P = 0.24
	C	156	3.482	0.068	3.349-3.616	110	3.520	0.072	3.378-3.663	96	3.389	0.075	3.242-3.535		

*I = Intervention; C = Control.

### Group cohesion

The results indicate no statistically significant group × time intervention effects F (2,421) = 1.39, p = 0.24). Analyses do, however, suggest a significant overall time effect with levels of cohesion increasing between baseline and post intervention for those in the intervention condition (p = 0.000) but this was not sustained at three month follow-up (Table 3).

### Alcohol-related HIV risks

Findings from the alcohol-related HIV outcome measure suggest no group × time intervention effect F (1,134) = 0.36, p = 0.548 (Table 4).

**Table 4:** Alcohol-related HIV risks baseline and 3 month follow-up*

Substance-related HIV risk**	**Intervention**		**Control**	
	**Baseline**	**3 month follow-up**	**Baseline**	**3 month follow-up**
	**N = 136**	**N = 80**	**N = 128**	**N = 79**
Mean	0.313	0.363	0.272	0.261
SE	0.051	0.066	0.048	0.059
95% CI	(0.222-0.421)	(0.245-0.501)	(0.187-0.376)	(0.162-0.392)
F-statistic	F (1,134) = 0.36			
P-value	0.548			

*Of those who consume alcohol.

**Variable analysed as a count variable.

### Willingness to utilise onsite-EAP

Although the data reflects a slight increase (in the predicted direction), from baseline to post intervention, in the likelihood of participants in the intervention condition using the EAP programme at their workplace, and a slight decrease for employees in the control condition, the effect was not sustained at 3 month follow-up F (2,334) = 0.77, p = 0.46 (Table 5).

**Table 5:** **Help-seeking behaviours at baseline, post intervention and at 3 month follow-up**

**Help seeking behaviours**	Group*	**Baseline**				**Post intervention**				**3-month follow-up**				**F-statistic**	**P-value**
		**N**	**Mean**	**SE**	**CI**	**N**	**Mean**	**SE**	**CI**	**N**	**Mean**	**SE**	**CI**		
*Willingness to use EAP*	I	155	0.676	0.063	0.543-0.786	117	0.681	0.067	0.537-0.797	89	0.596	0.079	0.436-0.738	F (2,334) =0.77	P = 0.46
	C	136	0.673	0.061	0.545-0.780	99	0.559	0.078	0.406-0.702	90	0.589	0.079	0.431-0.732		
*Likelihood of encouraging a co-worker to use the EAP*	I	143	0.753	0.056	0.753-0.056	114	0.796	0.053	0.796-0.053	86	0.720	0.070	0.720-0.070	F (2,350) =1.40	P = 0.24
	C	141	0.790	0.049	0.790-0.049	96	0.694	0.067	0.694-0.067	84	0.730	0.066	0.730-0.066		

*I = Intervention; C = Control.

### Recommending the EAP to co-workers

No significant group × time interaction was found between the intervention and control conditions at baseline and post-intervention and 3 month follow-up F (2,350) = 1.40, p = 0.24 in terms of recommending the EAP to co-workers (Table 5).

## Discussion

To our knowledge, this is the first RCT exploring the effectiveness of an alcohol and alcohol-related HIV prevention programme. Employees who received TA showed modest reductions in binge drinking from baseline to three month follow-up and going to work or calling in sick because of a hangover. This suggests that programmes such as TA that are based on the principles of social health promotion may be useful for addressing risky drinking practices in the workplace [18,20,25,37]. Although the results on the CAGE variable, including the climate and cohesion variables, measured in this study were small in comparison to the binge drinking and hangover variables, showing non-significant effect, they do provide a launch pad for strengthening and replicating future RCT studies on TA, especially in developing country settings.

Findings from the RCT analyses regarding associations with alcohol-related HIV risks suggest that the intervention had no effect on HIV risk. This can be attributed to the fact that this section of the instrument was plagued by poor response at baseline data collection. Although this improved at three month follow-up, it suggests that the baseline data was possibly an artefact of poor reporting but due to continuous reinforcement (by fieldworkers) in relation to encouraging honesty in answering questions, response to questions did improve later on in the study. This is not surprising since similar results were reported in a study where participants reported a resistance to disclosing HIV risks since they did not understand the purpose of the risk valuation [45]. In addition there are barriers, such as stigma, related to disclosure of not only sexual risk behaviour but any issue related to individual sexual health [46]. Lack of knowledge of HIV risks and the perception that one is not at risk may also serve as a contributing factor more so among middle aged adults [47]. Future studies replicating TA should address the shortcomings encountered in this study. It may be useful to explore possible contextual and other factors that act as possible barriers to persons answering alcohol-related HIV questions, particularly for this population who are largely male.

Although we anticipated that exposure to TA would significantly increase help-seeking behaviour, the overall TA effects on help-seeking were weak. The aim of TA is to encourage help-seeking behaviour through creating an understanding and awareness of the benefits of the EAP programme in the context of the company alcohol and drug policy. In this study, a great deal of uncertainty and wariness existed around the existing municipality’s in-house EAP policy, contributing to reluctance in utilising the EAP. Literature suggests that there are certain organisational factors that impede on utilisation of EAP services, such as perceptions around the neutrality and confidentiality of EAP services [48,49], manager support [3,48,49], stigmatisation [21], EAP service quality and programme awareness [48,50]. It is therefore essential to evaluate EAP favourability, remedy resistance and remove barriers to service use [49], prior to running a programme since referral remains the capstone of the programme.

Although the findings reported are encouraging it should be interpreted in the context of certain limitations. Although the investigators are confident that interventionists followed process and fidelity protocols in delivering TA, there may be factors that could have contributed to the inability of the study to generate certain hypothesised effects. For instance, level of participant engagement and participation in all of the modules were not measured using an evaluation checklist, instead participants were asked at the end to comment on their experience of the intervention. The use of session ratings is recommended in future replications of TA. Additionally, the interventionists were mostly female and this may have acted as a barrier since both divisions are male dominated. In addition, initial resistance to participate may have also impacted on willingness to be honest particularly at the start of the research study.

The sample used in this study was drawn from a single Metropole and may not be representative of or generalisable to all municipal employees within safety and security occupations. However, the settings and conditions under which the study was conducted are typical of municipalities located in South Africa as well as safety and security workplaces [38].

The study also made use of self-report questionnaires. Although recent research indicates that self-report surveys are equally as reliable as biological markers in assessing for alcohol and drug use [51], it is our view that the study may have benefitted from the inclusion of biological markers. Support for this assertion can be found in the extent of underreporting noticed in this study, particularly in relation to the use of drugs. A study conducted in 2003 found that the harder the drug, the less likely participants would be honest about their drug use possibly due to the stigma associated with the use of these drugs or fear of legal repercussions [52]. Further contributing to a general reluctance to answer questions on behaviours that are considered undesirable may also stem from a distrust of the research process or even fear that the researches would expose participants to management. Similar limitations were reported in studies conducted by Holcom and colleagues who add that such perceptions by employees do not emanate from the research team following incorrect research procedures but rather, a distrust of something that is considered new and also a distrust of a work system that is viewed with suspicion [53]. Additionally, contributing to a reluctance to self-disclose was the actual occupation (safety and security) the participants were employed in [38]. Within this profession there is a requirement to uphold certain behaviours and conduct, and substance abuse behaviours are considered contrary to this. Similar limitations were also reported on the substance-related HIV self-report questions which are consistent with other studies where resistance to disclosing HIV risks have been reported. It is clear that participants need to be assured of the confidentiality of the research process throughout the duration of the study process, but more particularly at the onset [38,45].

Attrition in this study was also relatively high at both post-intervention and at three month follow-up although missing values did not relate to participants dropping out of the study because of lack of interest but rather due to unplanned work requirements and other work commitments (see Figure 2). It should further be highlighted that there were no significant baseline differences on any of the variables used and analysis used to predict differences among employees who dropped out of the study versus those who completed the study were also not significantly different. While attrition in workplace studies are not unique [5,54] it is not ideal since it can reduce the numbers in the analysis which could have resulted in some statistical tests reaching significance. A review conducted by Schulte and colleagues also suggest that the stigma associated with receiving an alcohol-related intervention impacts significantly on the implementation of interventions in the workplace and may be a reason for the low-participation rates of risky drinkers in the workplace setting [28]. In fact Cook and Schlenger suggest that stigma could be described as ‘the most singular obstacle to workplace prevention’. The authors argue that the same company that embraces cholesterol screening sessions, weight management programmes and other health promotion programmes is probably more likely to shun programmes on alcohol or drug screening and, employees may also be resistant to attending such programmes. While, considerable advances have been made toward the general acceptance of substance abuse as a disease and therefore a treatable affliction; employees are rarely willing to take any action that might indicate that they could have a substance abuse problem [21]. Further fuelling low participation rates could be the mostly male sample of this study since risky drinking is more prevalent in males, who are generally more inclined to reject therapeutic interventions for mental health conditions [28].

Furthermore, alcohol use was measured using single item measures and the CAGE instrument. While the CAGE questionnaire was used because it has been validated extensively, and is short, simple and easy to answer, the study would have benefitted from the use of other standardised instruments that measure severity of substance abuse by providing cut-off scores. Examples of such screening tools are the Alcohol Use Disorders Identification Tool- C- FAST and the WHO ASSIST. Similarly, more reliable measures such as validated scale for examining alcohol-related HIV risks in this study sample are recommended.

## Conclusions

This intervention study was able to demonstrate a modest but statistically significant reduction in binge drinking and going to work with a hangover, and non-significant (but in the predicted direction) reductions in three other risk variables following the TA intervention. This provides encouraging evidence for interventions that address risky use of alcohol among employed persons. Although there were no changes to alcohol-related HIV risks, to our knowledge TA is the first evidence-based workplace prevention programmes to be tested in South Africa and the first RCT to assess’ changes in risky alcohol use and alcohol-related HIV risk among safety and security workers in this country. Further investigations are needed to address the limitations encountered and determine effect of TA on problem drinking in different working populations.
